# Establishing a Reference Dose–Response Calibration Curve for Dicentric Chromosome Aberrations to Assess Accidental Radiation Exposure in Saudi Arabia

**DOI:** 10.3389/fpubh.2020.599194

**Published:** 2020-12-15

**Authors:** Ghazi A. Alsbeih, Khaled S. Al-Hadyan, Najla M. Al-Harbi, Sara S. Bin Judia, Belal A. Moftah

**Affiliations:** ^1^Radiation Biology Section, Biomedical Physics Department, King Faisal Specialist Hospital and Research Centre, Riyadh, Saudi Arabia; ^2^College of Medicine, Alfaisal University, Riyadh, Saudi Arabia; ^3^Medical Physics Unit, Department of Oncology, McGill University, Montreal, QC, Canada

**Keywords:** dose-response curve, radiation exposure, chromosome aberration, biodosimetry, Dicentric Chromosome (DC), Dicentric chromosome assay (DCA), radiation emergency preparedness

## Abstract

In cases of nuclear and radiological accidents, public health and emergency response need to assess the magnitude of radiation exposure regardless of whether they arise from disaster, negligence, or deliberate act. Here we report the establishment of a national reference dose–response calibration curve (DRCC) for dicentric chromosome (DC), prerequisite to assess radiation doses received in accidental exposures. Peripheral blood samples were collected from 10 volunteers (aged 20–40 years, median = 29 years) of both sexes (three females and seven males). Blood samples, cytogenetic preparation, and analysis followed the International Atomic Energy Agency EPR-Biodosimetry 2011 report. Irradiations were performed using 320 kVp X-rays. Metafer system was used for automated and assisted (elimination of false-positives and inclusion of true-positives) metaphases findings and DC scoring. DC yields were fit to a linear–quadratic model. Results of the assisted DRCC showed some variations among individuals that were not statistically significant (homogeneity test, *P* = 0.66). There was no effect of age or sex (*P* > 0.05). To obtain representative national DRCC, data of all volunteers were pooled together and analyzed. The fitted parameters of the radiation-induced DC curve were as follows: *Y* = 0.0020 (±0.0002) + 0.0369 (±0.0019) ^*^ *D* + 0.0689 (±0.0009) ^*^ *D*^2^. The high significance of the fitted coefficients (*z*-test, *P* < 0.0001), along with the close to 1.0 *p-*value of the Poisson-based goodness of fit (χ^2^ = 3.51, degrees of freedom = 7, *P* = 0.83), indicated excellent fitting with no trend toward lack of fit. The curve was in the middle range of DRCCs published in other populations. The automated DRCC over and under estimated DCs at low (<1 Gy) and high (>2 Gy) doses, respectively, with a significant lack of goodness of fit (*P* < 0.0001). In conclusion, we have established the reference DRCC for DCs induced by 320 kVp X-rays. There was no effect of age or sex in this cohort of 10 young adults. Although the calibration curve obtained by the automated (unsupervised) scoring misrepresented dicentric yields at low and high doses, it can potentially be useful for triage mode to segregate between false-positive and near 2-Gy exposures from seriously irradiated individuals who require hospitalization.

## Introduction

Exposure to ionizing radiations poses an increasing threat to public health, from accidents involving medical radiological equipment failure, nuclear reactors, lost radioactive sources, and terrorist events incriminating radiation dispersal devices, radiation exposure devices, and improvised nuclear devices (INDs) (1). Regardless of whether radiation overexposure arises from disaster, negligence, or deliberate act, professionals working in the management of nuclear and radiological accidents are usually confronted with an array of complex issues when reacting to a radiation emergency (2). An important medical aspect is the estimation of the biological radiation dose received by the victims, in order to provide suitable dose assessment, medical triage, diagnoses, and treatment (3).

To provide diagnostic information to treating physicians, the effective medical management of suspected acute radiation overexposures includes recording dynamic medical data, performing appropriate radiation bioassays for dose estimation, and measuring radioactivity for dose reconstruction (4). These include observing and documenting radiation-induced prodromal signs and symptoms, obtaining initial blood sample for complete blood counts and white blood cell differentials, assessing physical dose directly from the accident scene and personal dosimeters if available, and sampling blood for radiation bioassays. This is important because in the cases of radiation accidents with estimated exposures greater than 1 Gy, treating physicians may expect a range of dose-dependent deterministic effects, including serious acute radiation syndromes (ARSs) arising in the following weeks and months (5). In these cases, dose estimation will help attending physicians to set an appropriate tactic when or before symptoms of ARS appear (6). On the other hand, in the cases of radiation exposures with doses of less than 1 Gy, dose estimation is informative because of the potential risk of developing late stochastic effects, such as cancer, which mainly require long-term follow-up of exposed individuals (7).

The critical element in dealing with the health consequences of accidental radiation exposure is to provide a reliable measure of the biologically absorbed dose in the victims. The so-called biodosimetry is the dose estimation after exposure to ionizing radiation by means of changes in biological endpoints, or biomarkers (8). In the cases of radiological accidents, whether physical dosimetry is not available or disputed, these biomarkers can be used to identify individual exposure cases. The basic principle of biodosimetry is to utilize biologic changes induced by ionizing radiation in body components of exposed individuals to estimate the radiation dose received. This absorbed dose can predict the clinically relevant response and the biological consequences of accidental radiation exposure. Ideally, the biologic changes should be, as much as possible, specific for ionizing radiation, and the biomarkers should be minimally affected by physiological or prior medical variations among people, including changes that might be caused by the stress and trauma from the radiation accident (9).

It is well-known that ionizing radiations produce several biological effects; some of them are quantifiable and thus can be used to estimate the radiation dose. Hence, research in the field of biodosimetry has advanced significantly, with expansion into the disciplines of genomics, proteomics, metabolomics, and transcriptomics, in addition to the classic cytogenetic approaches. However, when looking for a biological dosimeter, it is necessary to consider other characteristics such as reproducibility, cost, minimal-invasiveness, and potential for standardization. As the DNA is the critical biological target for ionizing radiation, methods analyzing lesions to cellular DNA constitute logic targets for biological dosimeter when they meet the required properties of biomarkers. Among the various radiation-induced lesions [including single-strand breaks and double-strand breaks (DSBs), base damage, and DNA-protein cross-links], the DSBs are the most interesting for biodosimetry. This is because DSBs misrepair can produce translocation between two broken chromosomes that generates chromosomal aberrations observable at metaphase (10). Actually, the cell-based analysis of DSBs and chromosomal aberrations, including the well-established dicentric chromosome (DC) assay (DCA), and the cytokinesis-block micronucleus assay, remain the most reliable for radiation injury assessment (11) and are the most advanced biodosimetry methods to quantify ionizing radiation doses received in exposed individuals (6).

Currently, the cytogenetic analysis of DCs observed in metaphases from peripheral lymphocytes is the routine technique used to assess doses in cases of radiation exposures. DCA stands alone as the corner stone “gold standard” biodosimetry assay, recommended by the International Atomic Energy Agency (IAEA) (12) and standardized by the International Organization for Standardization (ISO) with constant update (13). The DCA has many advantages, such as a high specificity to ionizing radiation, the slow DC decay with the possibility of assessing exposure even months after irradiation, the potential to detect partial body exposure, and its sensitivity to doses down to 20 mGy when scoring a few thousand metaphases (14). Consequently, many countries have established specialized cytogenetic biodosimetry laboratories as part of the radiation protection program and the first medical responders to radiological and nuclear threats (15–19). We have previously described the establishment of a cytogenetic biodosimetry laboratory in Saudi Arabia along with producing a preliminary calibration curve composed of four volunteers (20). Since then, we have gained experience in cytogenetic preparation and chromosomal aberrations scoring. Here we report the establishment of a final reference dose–response calibration curve (DRCC) for DCs induced by X-rays (320 kVp), prerequisite to assess radiation doses received in accidental radiation overexposure in Saudi Arabia.

## Materials and Methods

### Volunteers, Blood Samples, and Ethical Considerations

Ten healthy Saudi volunteers, aged between 20 and 40 years, were enrolled in this project. The study was approved by the Basic Research and the Ethics Committees of King Faisal Specialist Hospital and Research Centre (KFSHRC) institutional review board (KFSHRC, RAC#2170 005). Data related to the history of significant diseases, prior radiation exposure, smoking, or drug use were collected at the time of blood donation. Volunteers' data were anonymized with no donor's identifiable script. After signing the informed consent, each volunteer donates a one-time 20 mL peripheral blood sample, which was taken by routine laboratory venipuncture in heparinized tubes (Vacuette; Greiner Bio-One GmbH, Germany). The fresh whole-blood sample was visually checked for any potential clotting and then aliquoted into 10 × 25-mL cell culture flasks (2 mL each), transferred to 5% CO_2_ incubator, and kept at 37°C to stabilize for a short time before irradiation.

### X-Ray Irradiation

Whole-blood peripheral lymphocytes were irradiated with 320 kVp X-rays. Each blood aliquot was irradiated with a single X-ray dose of either 0, 0.10, 0.25, 0.50, 0.75, 1, 2, 3, 4, or 5 Gy. The X-RAD 320 (Precision X-ray, CT, USA) biological irradiator was used for irradiation at a maximum energy of 320 kVp, 2 mm Al filter, and 1 Gy/min of dose rate. The source-to-surface distance was 70 cm, with maximum field size of 30 × 30 cm (uniformity across field: 93–100%). The measured half value layer was 1.12 mm Cu. Flasks containing blood samples were positioned horizontally in the middle of the irradiation field and were irradiated sequentially. The deviation between the preset dose and the delivered dose was within 3.1% as measured using calibrated ionizing chamber (PTW, Freiburg, Germany) and checked with GAFCHROMIC film (EBT2 model) as described previously (21).

### Whole-Blood Lymphocytes Culture

After 2 h of whole-blood incubation at 37°C following irradiation, 18 mL of prewarmed complete RPMI-1640 medium (including l-glutamine; Sigma–Aldrich, USA) and complemented with 15% fetal bovine serum (Hyclone, ThermoScientific, USA) and 1% of 100 × penicillin–streptomycin antibiotic solution (100 IU and 100 μg per mL; respectively; Sigma–Aldrich) were added to each flask along with 400 μL phytohemagglutinin (Remel Europe Ltd., ThermoScientific, USA) to stimulate lymphocytes division. Flasks of blood culture were incubated at 37°C, 5% CO_2_ atmosphere, for 48 h as recommended by the EPR-Biodosimetry 2011 (12), and optimized to capture first-division metaphases. Colcemid (Irvine Scientific, CA, USA) was added at a final concentration of 0.10 μg/mL, for the last 4 h to arrest lymphocyte cell division at metaphase.

### Hypotonic Treatment and Cell Fixation Procedures

Lymphocyte cell cultures were centrifuged at 1,100 rpm (200 g) for 8 min in 50-mL tubes, and the supernatant was gently removed. Cell pellet was resuspended; 10 mL of fresh hypotonic solution (0.075 M KCl) was gradually added and incubated at room temperature (RT) for 12 min. Soft fixation was performed by smoothly adding 2 mL of fresh fixative (3:1 methanol/acetic acid) and let to stand at RT for 10 min. Cells were then centrifuged, pellet was resuspended, and fresh fixative (10 mL) was added and incubated for 10 min at RT for 2 cycles. The fixed cells were stored overnight at −20°C prior to slides preparation.

### Giemsa Staining and Slide Preparation

Fixed lymphocytes were centrifuged (200 g) for 8 min and resuspended in an adequate volume of fixative to obtain a cloudy preparation to achieve suitable concentration of cells. To prepare metaphase spreads, 40 μL was pipetted and dropped on a moistened precleaned microscope slide (Superfrost; Fisher Scientific, Waltham, MA, USA). The spreads were dried on a slide warmer set at 40°C. Staining was performed in a coplin jar using a solution of 10% Giemsa in phosphate buffer (4 mL Giemsa in 36 mL PBS, pH 6.8) for 10 min. The slides were rinsed in distilled water, air-dried, mounted with Eukitt medium (Fluka; Sigma–Aldrich), covered with a coverslip, and sealed.

### Metaphases and Dicentric Chromosome Aberrations Scoring

The automated Metafer5 system (MetaSystems, Altlussheim, Germany) was used for the autocapture of metaphases, which include finding, image acquisition, storing, and relocation. This motorized microscope system allows to autoprocess up to 80 slides with the robotic SlideFeeder x80 module. Depending on cell density, at least three slides were loaded for each data point, and after initial image focus and alignment adjustment, the systems ran unattended overnight or over the weekend. Scoring of DC aberrations was carried out by exporting the files of the stored images into the DCScore image analysis system (MetaSystems). This module identifies and uniquely scores DCs using a trainable classifier preset from international collective data and tuned for each individual biodosimetry laboratory. The system classifies metaphases as containing 0, 1, 2, 3, 4, or 5 dicentrics, depending on the number of DCs found. The raw *automated scoring* (unsupervised), as set by the manufacturer, provides results with a certain inherent error related to the nature of the image analysis and the quality of metaphases and DCs in the cytogenetic preparation.

The *assisted scoring* is a semiautomated (supervised) counting that involved human verification and correction. It is conducted by reexamining the images of the automated electronic scoring files to determine the validity of the scores (elimination of false-positives and inclusion of missed true-positives). The x- and y-stage coordinates were used to allow metaphase relocation and reexamination when required. Validation steps were conducted by staff where images were reviewed for adequacy of both metaphases and DC scoring, and results were corrected accordingly. Only complete metaphases with nearly 46 discernable centromeres were included in the analysis. Other aberration types, including ring chromosomes and acentric fragments, were manually tracked at this stage wherever observed. To comply with the IAEA recommendations, we aimed to scan thousands of metaphases for each point, which was often achievable at the lower radiation doses. At higher doses, we targeted, as much as possible, to score at least 100 DCs. Three to six slides were scanned per data point, and results were pooled to acquire a sufficient number of metaphases or dicentrics (≥100 DCs for the high radiation doses) for proper data analysis.

### Statistical Analysis

The analysis of DC aberrations frequency and the construction of the DRCCs followed the methods described in details in the IAEA Technical Report 405 (10), EPR-Biodosimetry 2011 (12), and described previously by our group (20). Briefly, the yield (*Y*) of DCs was calculated by dividing the total number of dicentrics found by the entire number of metaphases counted per radiation dose. The variance (Var), standard error (SE), dispersion index (DI), and *U*-test were computed, taking into consideration Poisson distribution of the number of dicentric per metaphases. This stipulates that ideally the values of the standard *U-*test are included between ±1.96. Thus, if the magnitude of the *U*-value is out of that range, the dispersion of dicentrics is considered significant at the 5% level. The Dose Estimate software was used for computation, including correction when variance (Var) exceeded mean DC yield (*Y*), correction for overdispersion as needed, and conduction of goodness-of-fit test (22). Subsequently, the dose–response relationship was established for DCs. As sufficient number of degrees of freedom (DF) is required for adequate curve fitting, 10 different radiation doses were included in the range between 0 and 5 Gy according to ISO guidelines (13). Data were fitted using the linear–quadratic dose–response curve (*Y* = *C* + α*D* + β*D*^2^) by the method of maximum likelihood using Dose Estimate and the free statistical software package R (version 3.6.3). The coefficients of the fitted curves (the intercept *C*, the linear α, and the quadratic β components) were derived for each of the 10 individuals and for respective pooled donors' data. The 95% confidence interval of the fitted curves was computed assuming Poisson distribution of the DC yields. A χ^2^ test for homogeneity was used to test for differences between data sets in observed numbers of dicentrics in scored cells. For differences in DCs yields between groups of volunteers, the parametric one-way repeated-measures analyses of variance or the non-parametric Friedman repeated-measures analyses of variance on ranks tests were performed using SigmaPlot software (version 13; Systat Software Inc., San Jose, CA, USA). *P* < 0.05 was considered statistically significant.

## Results

The 10 healthy volunteers had no noticeable history of significant diseases, radiation exposure, or drug use at the time of blood donation. Four donors were active smokers with no obvious effects on DC yield. There were three females and seven males. Ages ranged between 20 and 40 years (median = 29 years). Yields of DCs were determined in first-division metaphases obtained from optimized 48-h blood cultures (12). The automated Metafer5 system was used to autocapture metaphases, detect, and score DC aberrations. The important validation step makes use of staff expertise in reviewing metaphases and, where needed, ascertain dicentrics by observing other aberrations. The DCs were frequently associated with the presence of acentric (Ac) fragments and sometime rings (R), which were spotted manually during the validation step as they are not scored by Metafer system. However, the documentation of chromosomal aberrations other than dicentrics is not exhaustive as they are not used to construct the DCs dose–response relationship of biodosimetry. While the main reported DC results are those obtained with the assisted (semiautomated or supervised) scoring, the Metafer-generated raw DC results of automated (unsupervised) scoring were also presented and discussed in relevance to their potential application in triage mode in the cases of mass-causality accidents.

**Figure 1:**
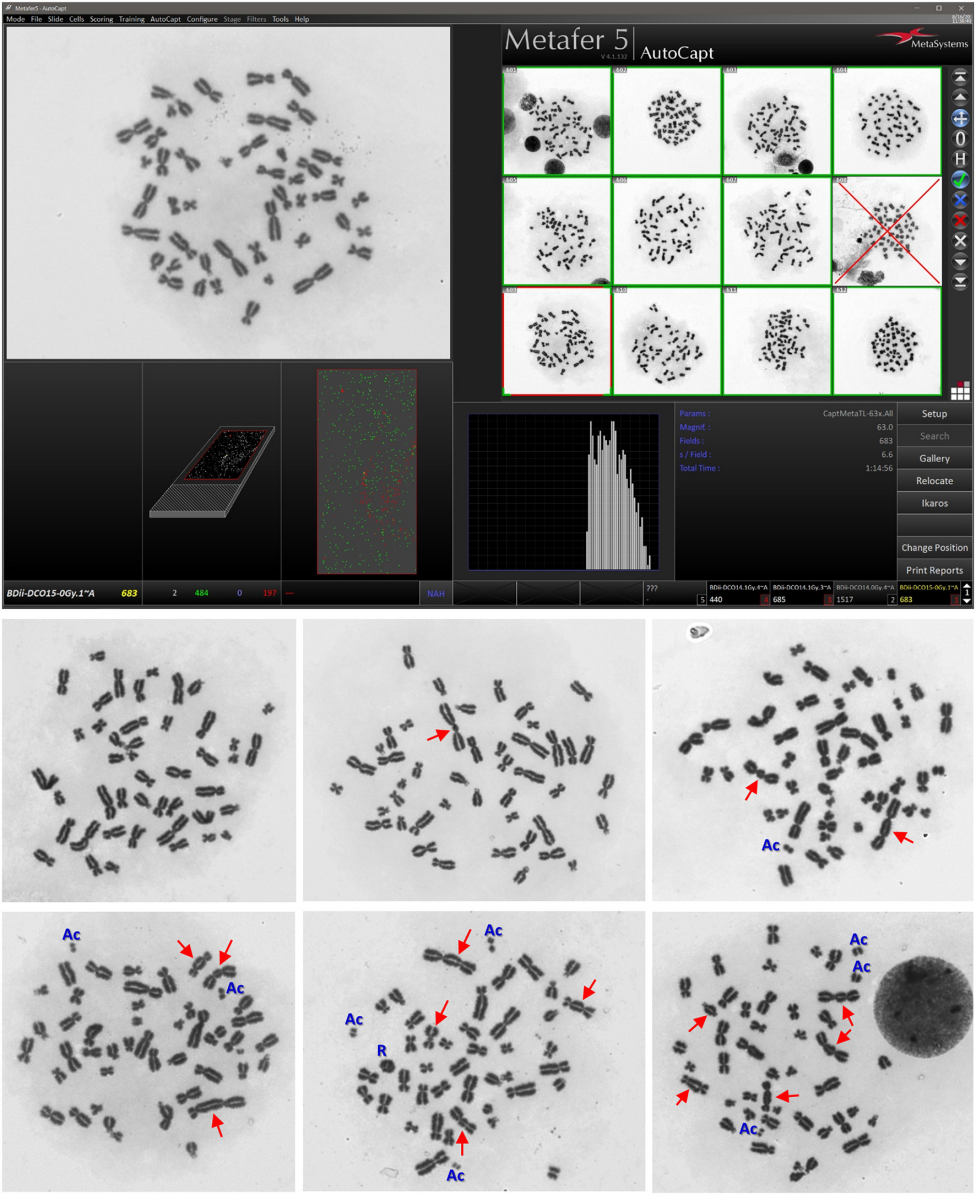
Representative examples of Metafer5 interface screenshot (**upper**) along with normal metaphase, and metaphases with dicentrics (DCs; arrows) and other observed aberrations, such as acentric fragments (Ac) and ring (R) (**lower**). The average number of dicentric per metaphase (i.e., DC yield) and the appearance of other aberations increase with increasing radiation dose received by the lymphocytes in the blood samples.

**Figure 2:**
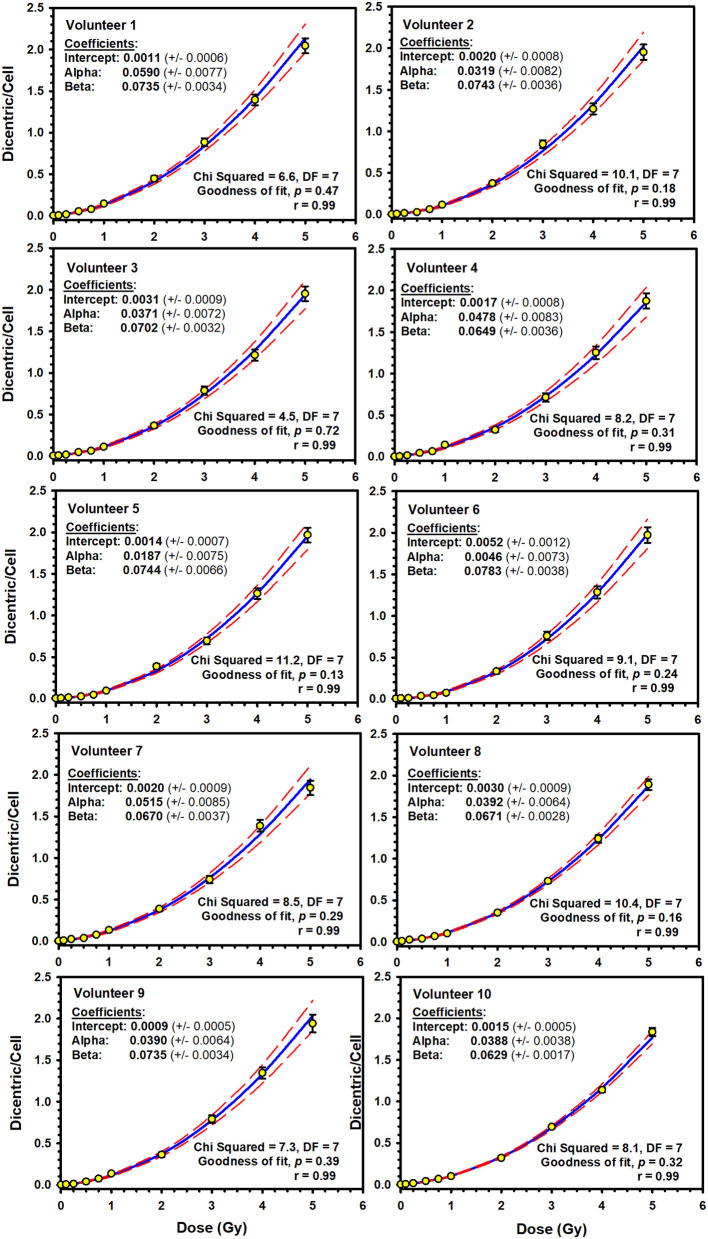
Individuals' linear–quadratic dose–response curves (solid lines) for dicentric chromosome aberrations induced by 320 kVp X-rays in lymphocytes derived from 10 Saudi volunteers. Data points represent the yield of dicentric per metaphase scored using assisted (supervised) mode. Dashed lines indicate the 95% confidence limits calculated assuming Poisson distribution of the dicentrics data. Error bars represent the standard errors of the dicentric yield.

**Figure 3:**
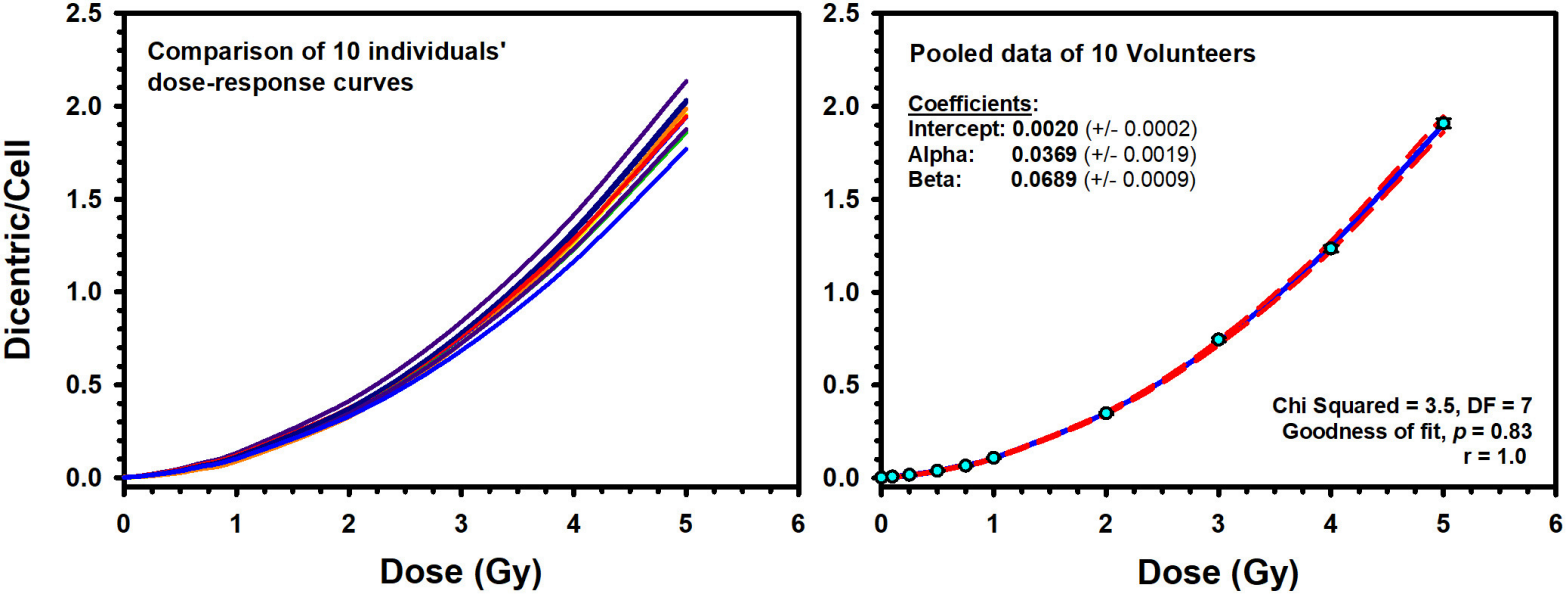
**Left**: Comparison of the individuals' dose–response curves for dicentric chromosome aberrations induced by 320 kVp X-rays in lymphocytes derived from 10 Saudi volunteers. **Right**: The reference dose–response calibration curve (solid line) generated from the pooled data of the 10 volunteers. Dashed lines indicate the 95% confidence limits calculated assuming Poisson distribution of the dicentrics data. Error bars represent the standard errors of the dicentric yield.

Illustrative examples of Metafer5 interface and normal, DCs, and other aberrations containing metaphases are presented in Figure 1. The data of each individual volunteer, obtained with the assisted (supervised) scoring, are listed in the Supplementary Material 1. The resulting individuals' DC dose–response curves, along with the curve fitting parameters, are shown in Figure 2. The goodness of fit was satisfactory (*P* > 0.05, i.e., no significant lack of fit) in the 10 individuals. Note that all data were corrected for both, variance (Var) exceeding mean DC yield, and SE for overdispersion, as needed, using the Dose Estimate software. The results show that the DC yields increase quickly with increasing dose in a trend that is similar in the 10 individuals. The comparison between the fitted curves displayed little variation between the 10 individuals (Figure 3). For example, the fitted DC yield at the highest radiation dose of 5 Gy showed a narrow range between 1.8 and 2.1 (mean = 1.95) dicentric/cell.

To construct a national reference DRCC representative of the population in Saudi Arabia, the data of the 10 individuals were pooled together and analyzed. The pooled DC data, obtained by assisted (supervised) scoring, are shown in Table 1, which enlists the number of metaphases analyzed, dicentrics observed, distribution of dicentrics, the average number of DCs per metaphase (DC yield), and related statistics. Obviously, there was a sharp decrease in the recorded number of metaphases with increasing radiation doses, which was offset by relative rise in the number of DCs observed. Hence, while tens of thousands of metaphases were recorded at the lower radiation doses, thousands of DCs were scored at the higher doses. The DI was about 1 at the different radiation doses, and its normalized unit (*U*-test) was included in the range of ±1.96, indicating good compliance with Poisson distribution. In total, there were 21,963 DCs found in 147,100 analyzed metaphases (average yield of 0.15 DC per metaphase). The DC background level determined by the examination of 37,954 metaphase spreads of unirradiated blood samples was ~2 DCs per 1,000 metaphases. After the exposure to increasing radiation doses from 0.10 to 5.0 Gy, the number of DCs increased including some metaphases exhibiting 2, 3, 4, and even 5 (the maximum number recorded by Metafer system) DCs (Table 1). The increasing dose was associated with a steep decline in the number of scorable metaphases (about 10-fold reduction; from 24,220 for 0.10 Gy down to 3,047 for 5 Gy). Conversely, the yield of DCs had sharply increased with increasing dose from 0.006 to 1.908 dicentric per metaphase. The fitted DRCC for DCs constructed from the pooled data is presented in Figure 3. The curve exhibits a characteristic linear–quadratic shape, and all data points are included within the 95% limits of the confidence interval, which is calculated assuming Poisson distribution. The fitted coefficients were: *Y* = 0.0020 (±0.0002) + 0.0369 (±0.0019) ^*^ *D* + 0.0689 (±0.0009) ^*^ *D*^2^). The goodness of fit for the curve of DC induction (weighted χ^2^ = 3.51, DF = 7, *P* = 0.83; i.e., there is no significant lack of fit) and the *p*-values for coefficients (*z*-test, *P* < 0.0001) indicate an excellent fitting.

**Table 1:** Yield and intercellular distribution of dicentric chromosomal aberrations induced *in vitro* in peripheral blood lymphocytes by X-rays exposure.

**Dose (Gy)**	**No. metaphases**	**No. dicentrics**	D0^*^	D1^*^	D2^*^	D3^*^	D4^*^	D5^*^	***Y***	**SE**	**DI**	***U*-value**
0	37,954	76	37,878	76	0	0	0	0	0.002	0.000	1.00	−0.27
0.1	24,220	156	24,064	156	0	0	0	0	0.006	0.001	0.99	−0.71
0.25	19,789	317	19,474	313	2	0	0	0	0.016	0.001	1.00	−0.33
0.5	16,322	625	15,710	599	13	0	0	0	0.038	0.002	1.00	0.30
0.75	14,367	933	13,459	885	21	2	0	0	0.065	0.002	0.99	−0.59
1	13,028	1,408	11,690	1,278	51	8	1	0	0.108	0.003	1.01	0.57
2	8,212	2,860	5,813	1,977	387	31	4	0	0.348	0.007	1.00	0.28
3	5,645	4,198	2,674	2,017	725	191	32	6	0.744	0.011	0.99	−0.27
4	4,516	5,575	1,293	1,662	1,019	348	139	55	1.235	0.017	1.00	0.11
5	3,047	5,815	407	986	733	495	252	174	1.908	0.025	0.97	−1.04

*Results of pooled data of 10 healthy Saudi blood volunteers*.

*U-value: a U-value between −1.96 and +1.96 indicates a Poisson distribution*.

* *Number of metaphases with 0, 1, 2, 3, 4, and 5 dicentrics, respectively*. *No. metaphases, number of cells in metaphase assessed; No. dicentrics, total number of dicentrics found in the metaphases assessed; Y, yield of dicentrics, i.e., the number of dicentrics per metaphase (cell); SE, standard error on yield (Y); DI, dispersion index*.

**Figure 4:**
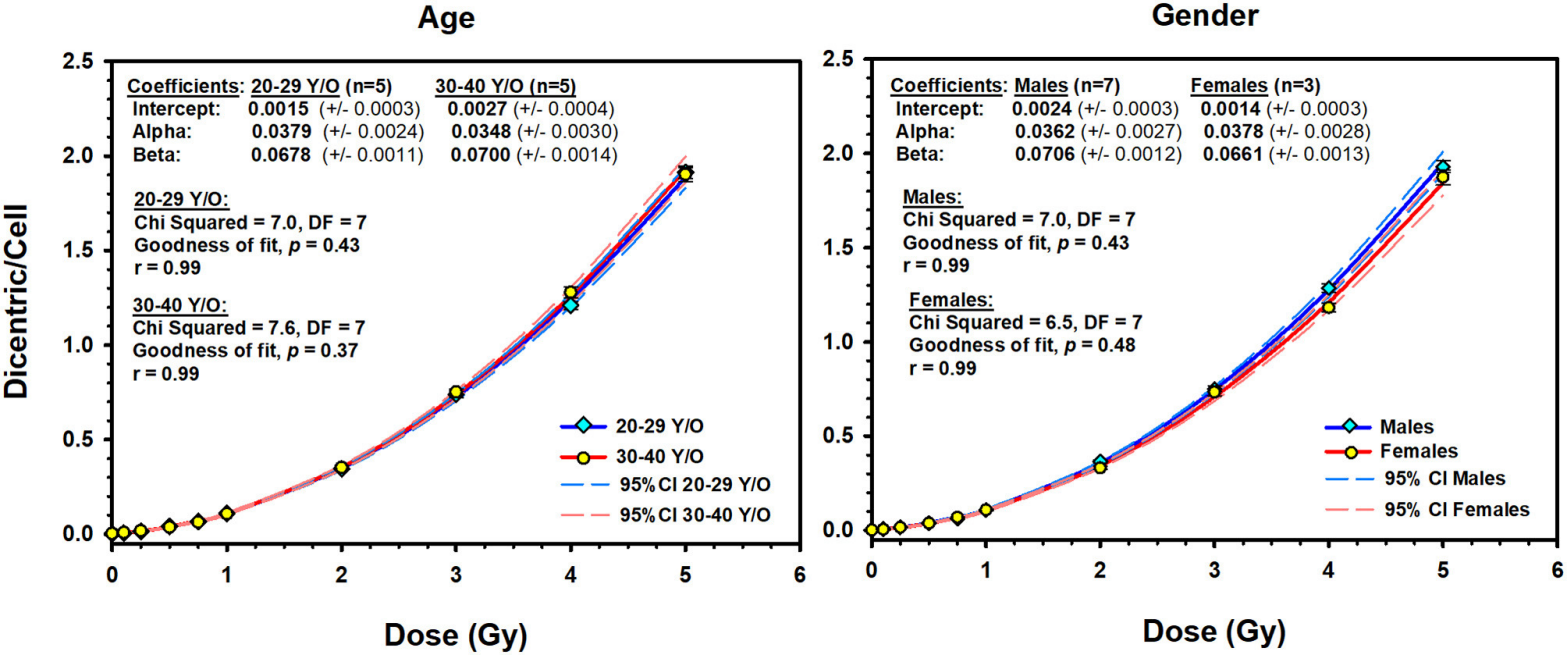
The effect of age (**left**) and sex (**right**) on the dose–response curves (solid lines) of dicentric chromosome aberrations in 10 Saudi volunteers. Dashed lines are the 95% confidence limits calculated assuming Poisson distribution of the dicentrics data. Error bars represent the standard errors of the dicentric yield.

In addition, we have compared the potential influence of age and sex on the DCs dose–response curve. For age, we have split the 10 volunteers, according to the median age of 29 years, into 2 groups (20–29 years old, *n* = 5 individuals; 30–40 years old, *n* = 5 individuals). The resulting dose–response curves were almost identical in this group of young adults (Figure 4). The Friedman repeated-measures analysis of variance on ranks confirmed this observation by showing no significant difference in the median number of DC yields (0.088 vs. 0.085) between the two groups (*P* = 0.75). The comparison between males (*n* = 7) and females (*n* = 3) showed slight decrease in DC yields in females [mean = 0.44, standard deviation (SD) = 0.64] compared to males (mean = 0.46, SD = 0.67) that did not reach statistical significance (one-way repeated-measures analysis of variance, *P* = 0.09).

**Figure 5:**
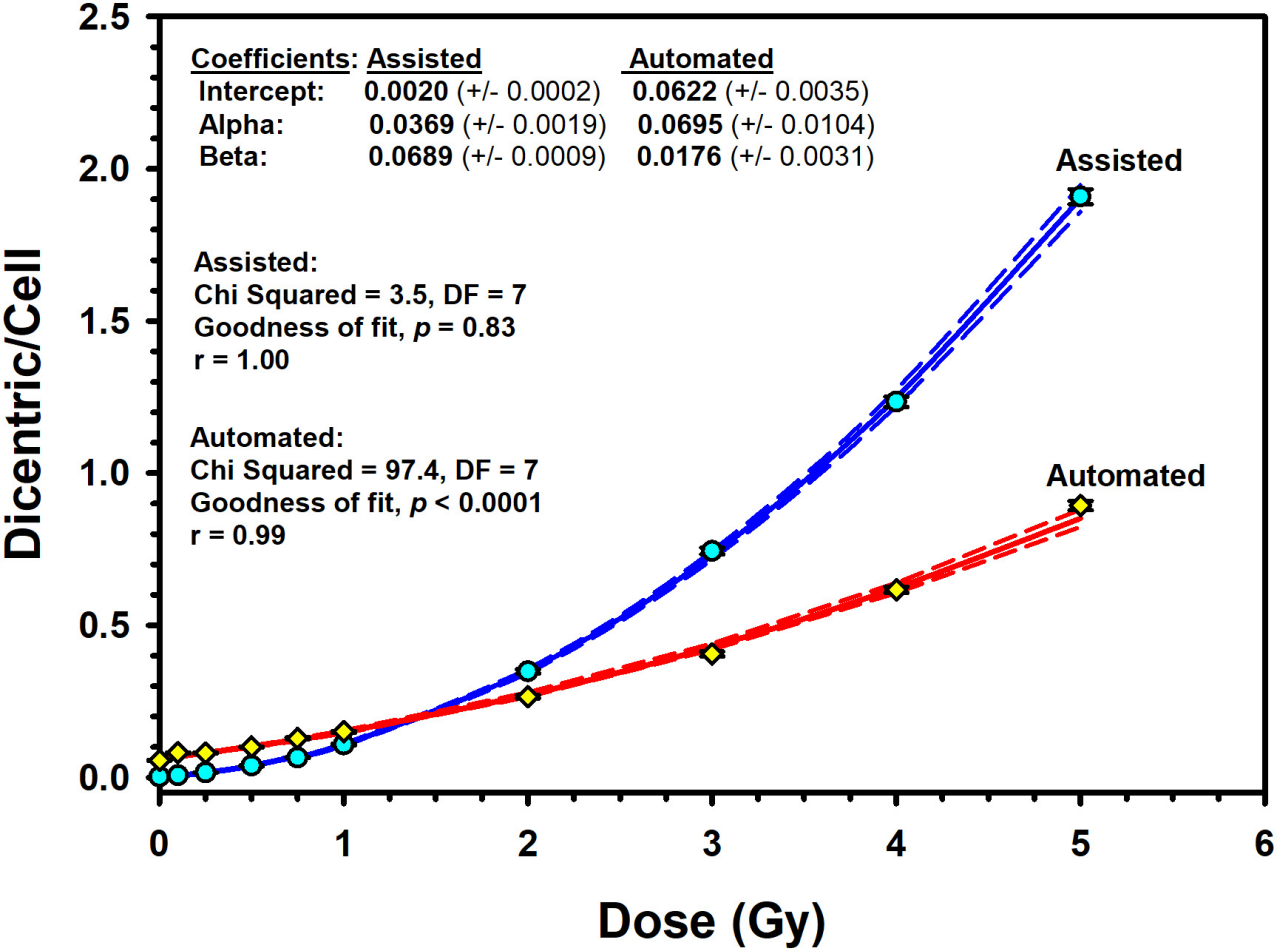
Assisted (supervised) and automated (unsupervised) reference dose–response calibration curves (solid lines) for dicentric chromosome aberrations induced by 320 kVp X-rays in lymphocytes derived from 10 Saudi volunteers. Data points represent the yield of dicentric per metaphase scored. Dashed lines indicate the 95% confidence limits calculated assuming Poisson distribution of the dicentrics data. Error bars represent the standard errors of the dicentric yield.

The results of the pooled data of the Metafer5 automated (unsupervised) scoring are shown in Table 2. Overall, in unsupervised scoring, there were obviously higher number of metaphases and DCs scored compared to assisted scoring. In total, there were 25,283 DCs in 167,242 metaphases autocounted; however, the average yield of DCs per metaphases (0.15) remained similar to assisted scoring. More importantly, there was overestimation of DCs at low radiation doses compared with higher doses with a relative overdispesion of DC as indicated by the *U*-test (Table 2). The resulting automated DRCC is given in Figure 5. The fitted coefficients for automated scoring were as follows: *Y* = 0.0622 (±0.0035) + 0.0695 (±0.0104) ^*^ *D* + 0.0176 (±0.0031) ^*^ *D*^2^). The goodness of fit for the fitted curve (weighted χ^2^ = 97.4, DF = 7, *P* < 0.0001) indicates significant lack of fit. The *p*-values for coefficients (*z*-test) were all significant, *P* < 0.001. In comparison with assisted scoring, there was an overestimation of DC yields under 1 Gy and underestimation greater than 2 Gy, leading to an overall steep decrease in DC yields at high doses (Figure 5).

**Table 2:** Metafer automated scoring of dicentric chromosomal aberrations induced *in vitro* in human lymphocytes by X-rays exposure.

**Dose (Gy)**	**No. metaphases**	**No. dicentrics**	D0^*^	D1^*^	D2^*^	D3^*^	D4^*^	D5^*^	***Y***	**SE**	**DI**	***U* value**
0	40,792	2,278	38,550	2,209	30	3	0	0	0.056	0.001	0.98	−3.08
0.1	27,245	2,192	25,151	2,004	82	8	0	0	0.080	0.002	1.02	1.90
0.25	22,665	1,819	20,936	1,642	84	3	0	0	0.080	0.002	1.02	2.35
0.5	18,160	1,819	16,454	1,601	97	8	0	0	0.100	0.002	1.03	3.14
0.75	16,605	2,142	14,605	1,869	121	9	1	0	0.129	0.003	1.01	1.35
1	14,641	2,200	12,619	1,862	142	18	0	0	0.150	0.003	1.03	2.40
2	9,943	2,636	7,641	2,021	234	42	4	1	0.265	0.005	1.03	2.39
3	7,137	2,898	4,766	1,895	437	28	10	1	0.406	0.008	1.00	0.12
4	6,076	3,746	3,306	1,912	763	74	19	2	0.616	0.010	0.98	−1.04
5	3,978	3,553	1,612	1,541	547	206	60	12	0.893	0.015	1.03	1.48

*Results of pooled data of 10 healthy Saudi blood volunteers*.

*U-value: a U-value between −1.96 and +1.96 indicates a Poisson distribution*.

* *Number of metaphases with 0, 1, 2, 3, 4, and 5 dicentrics, respectively*.

*No. metaphases, number of cells in metaphase assessed; No. dicentrics, total number of dicentrics found in the metaphases assessed; Y, yield of dicentrics, i.e., the number of dicentrics per metaphase (cell); SE, standard error on yield (Y); DI, dispersion index*.

## Discussion

The establishment of biodosimetry resources is critical for public health, medical planning, and response to events of individual or mass casualties involving radiological or nuclear incidents (23). The search for proper radiation biomarkers is gaining momentum in recent years because of the increased risks of accidental radiation overexposure and maleficent acts (24). Although many candidate markers (genomics, proteomics, metabolomics, and transcriptomics) are in the pipeline of development, the cytogenetic approaches remain the most widely used biomarkers for radiation exposure. In particular, the well-established DCA continues to be the gold standard of biodosimetry (12). In addition, the DCA persists as the cornerstone for retrospective dosimetry in acute and recent radiation accidents after whole-body or partial-body exposure (22).

The main aim of this study was to establish a reference DRCC for DC, representative for people in Saudi Arabia using the cytogenetic expertise gained in our radiation biology laboratory. Most likely, radiological accidents are anticipated to happen because of external exposure to low LET radiation. Therefore, we set up to establish the national DRCC for DCs induced by X-rays as prerequisites to provide first-responder capabilities to assess radiation doses received in accidental exposures. Another planned development includes other types of ionizing radiations and a broader spectrum of cellular and molecular radiation biomarkers currently in active research (11).

In this study, we report the establishment of a national DRCC for DCs induced by X-rays of 320 kVp. The study comprised blood samples from 10 healthy volunteers (seven males and three females) aged between 20 and 40 years. The donors' median age was 29 years. This average age is representative of the active workforce who may well be at risk of accidental radiation exposure. The individuals' dose–response curves showed that the yield of DCs increased with dose in a linear–quadratic manner (Figure 2). The goodness of fit was satisfactory with no significant lack of fit (*P*-values were included between 0.13 and 0.72; Figure 2). Although each blood sample showed an individual dose–response curve, there were little differences between the 10 volunteers (Figure 3). At the highest radiation dose of 5 Gy, where the largest variations were seen, the fitted DC yields were included in a narrow range between 1.8 and 2.1 (mean = 1.95, *SD* = 0.10) DCs per metaphases. In addition, a homogeneity test on the actual cells/aberrations' counts showed no significant difference between the 10 volunteers (χ^2^ = 6.8, DF = 9, *P* = 0.66). Although individual variations cannot be totally excluded, these are small and within experimental variations as has already been seen in interlaboratory comparisons (25).

An interesting observation in this study, frequently ignored in the literature, is to underline that there were no differences in DC yields between age or sex groups in this small cohort of young adults (Figure 4). The comparison between the age groups (20–29 vs. 30–40 years) showed similar dose–response curves that were confirmed by the absence of significant difference in the median number of DC yields between the two groups (*P* = 0.75). In addition, homogeneity test showed that the background level of DCs did not differ significantly between the two age groups (χ^2^ = 3.3, DF = 1, *P* = 0.07), neither between the 10 individuals (χ^2^ = 15.8, DF = 9, *P* = 0.07). In fact, age was mainly reported to be an influencing factor for chromosomal translocations and micronuclei particularly at a much higher age (12). The comparison between males (seven volunteers) and females (three volunteers) showed basically the same conclusion. Although females showed a slight decrease in the mean number of DC yields (mean = 0.44, *SD* = 0.64) compared to males (mean = 0.46, *SD* = 0.67), the difference was not statistically significant (*P* = 0.09), generally leading to comparable dose–response curves (Figure 4). Thus, the 10 volunteers showed a mostly comparable DC yield induced by X-rays in the peripheral blood lymphocytes. This has essentially produced comparable linear–quadratic dose–response curves, characteristic of low LET radiation (Figure 3). Nevertheless, the sample size in this study (10 volunteers) is underpowered to detect subtle variations in DC yields between individuals and age and sex groups. While testing more samples of larger number of volunteers is required to confirm this conclusion, it nevertheless suggests low dicentrics variability among individuals, which implies that the data can be pooled and generalized to the related population.

The pooled assisted scoring results of the 10 volunteers have considerably produced a set of expanded data to improve reliability and representativity of the population (Table 1). This is in agreement with the IAEA recommendations on maximizing the number of metaphases scored and also as commonly practiced in biodosimetry field (26). In total, there were 21,963 DCs scored in 147,100 metaphases counted. This is fairly large number that exceeds other studies and gives strength to the results (12). In compliance with Poisson distribution, the DI was about 1 at the different radiation doses, and its normalized unit (*U*-test) was included in the range of ±1.96. The yield of DCs steadily increased with increasing radiation dose from 0.10 to 5 Gy. The lower dose tested of 0.10 Gy showed a yield (0.006 dicentric/cell) that is distinguishable from the background level (0.002 dicentric/cell). A homogeneity test indicates a statistically significant difference (χ^2^ =78.1, DF = 1, *P* < 0.0001). Therefore, the 0.1 Gy can be considered the lower detection limit tested (Table 1). Additional improvement can, however, still be brought about by testing lower doses and potentially improving dicentrics background level of nearly 1 dicentric per 1,000 metaphases (12, 26, 27).

**Figure 6:**
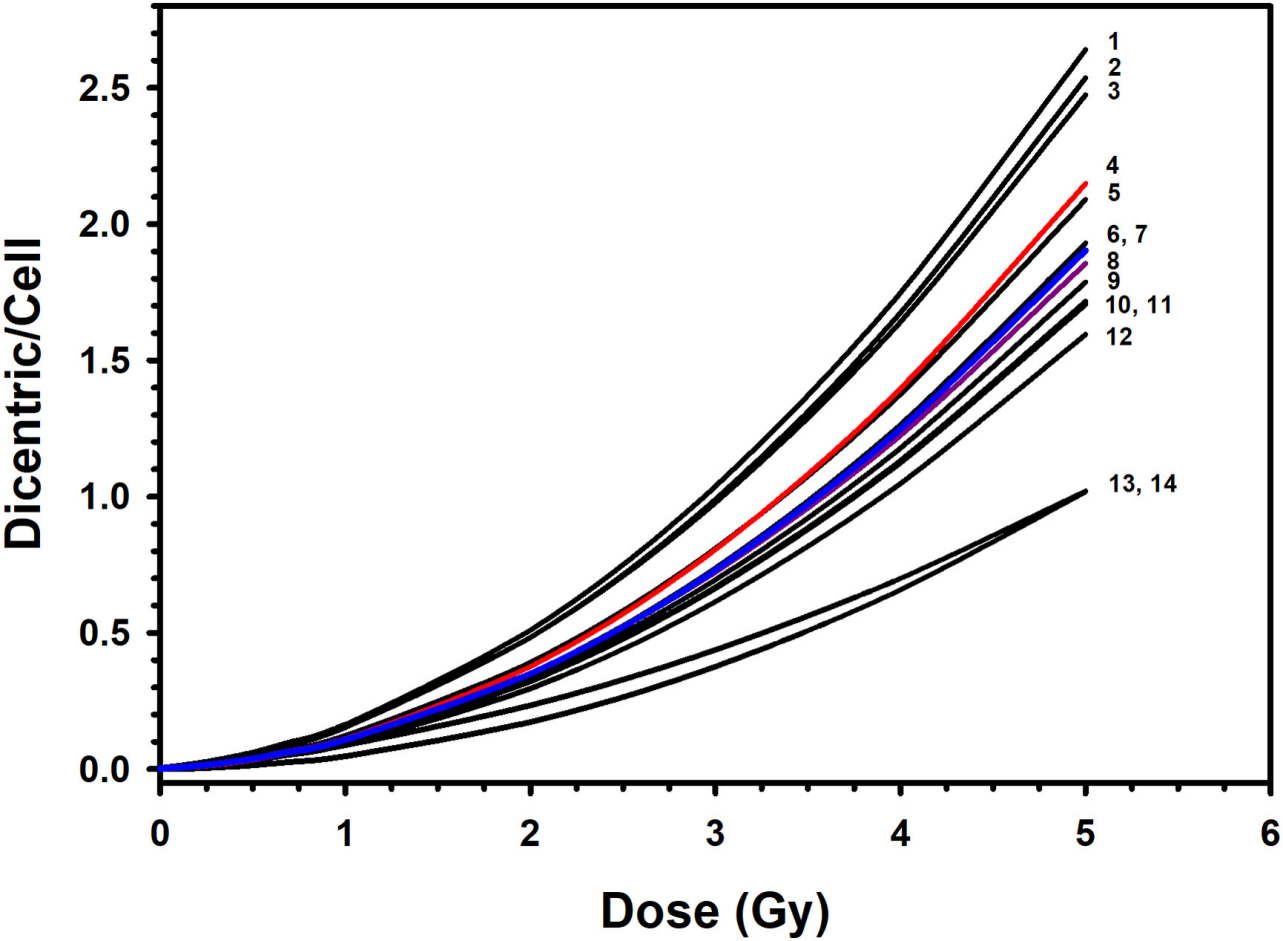
Comparison of the published dose–response calibration curves of dicentric chromosome aberrations induced by X-rays: 1. Germany, 90 kVp at 0.1 Gy/min (in (26); 2. Italy, 100 kVp at 1 Gy/min (30); 3. Canada, 250 kVp (29); 4. Preliminary dose–response curve in Saudi Arabia, 320 kVp at 1.33 Gy/min (20); 5. Germany, 250 kVp at 1 Gy/min (in (26); 6. The Netherlands, 100 kVp at 0.4 Gy/min (31); 7. Saudi Arabia, this study, 320 kVp at 1 Gy/min; 8. England, 250 kVp (28); 9. Germany, 240 kVp at 1 Gy/min (26); 10. Serbia, 250 kVp (33); 11. Germany, 220 kVp at 0.5 Gy/min (34); 12. Spain, 180 kVp at 0.27 Gy/min (in (26); 13. United States, 250 kVp at 1 Gy/min (32); 14. Indonesia, 122/250 kVp at 0.17 Gy/min (19).

The resulting reference DRCC combined from the 10 volunteers exhibited a classic linear–quadratic shape (Figure 4). The fitted national radiation-induced DC aberrations curve [*Y* = 0.0020 (±0.0002) + 0.0369 (±0.0019) ^*^ *D* + 0.0689 (±0.0009) ^*^ *D*^2^] is representative of the population for both males and females. Along with the high significance of the fitted C, α and β coefficients (*z*-test, *P* < 0.0001), the close to 1.0 *p-*value of the Poisson-based goodness of fit (χ^2^ = 3.51, DF = 7, *P* = 0.83) indicates clearly excellent fitting of the data with no trend toward lack of fit (12). The comparison between our dose–response relationship for DC induction with those from similar published studies that used X-ray irradiation (19, 26, 28–35) showed quite large range of DC yields (Figure 6). These interlaboratory differences could emanate from the energy of the X-ray irradiation used, the dose rate, technical or methodical variances, metaphases and DC scoring criteria, and the professional experience of the scorers. For examples, although most authors used X-rays of 220–250 kVp (26, 28, 29, 32–34), others used 100–180 kVp (19, 30, 31, 35). In addition, the dose rate varied from 1 Gy/min (26, 30, 32), to about 0.5 Gy/min (31, 34), to 0.27 Gy/min or less (19, 35) (Figure 6).

As a matter of fact, the wide variations of more than 2-fold in DC yields between various laboratories preclude the likelihood of using a dose–response curve established in a particular laboratory to estimate radiation doses in other biodosimetry test centers. For example, while the DC yield induced by 2-Gy dose ranged in other populations between 0.17 and 0.51 (mean = 0.36; *SD* = 0.10), it was 0.35 in our laboratory, which is in range with cytogenetic radiosensitivity of other populations. Comparatively, the reference DRCC in Saudi individuals is positioned in the middle of those published in other populations (Figure 6). Of particular note, our curve is very close to that published by Lloyd and colleagues (28), with both of them falling in the middle of all other published curves. Although the comparison with the previously published preliminary curve [*Y* = 0.0017 (±0.0004) + 0.0260 (±0.0046) × *D* + 0.0807 (±0.0024) × *D*^2^] (20) indicates slight reduction in the mean DC yields (0.49 vs. 0.45 dicentric/cells), the current results represent more refined curve computed from larger set of data. Such refinement is witnessed by the improvement in the minimum resolvable dose from 0.12 to 0.09 Gy (for six aberrations) between the preliminary and the up-to-date reference dose–response calibration curves; respectively. Therefore, this accurate reference DC DRCC can be applied to assess radiation overexposure and estimate the radiation doses received in cases of suspected accidental irradiation. For instance, a radiation dose received in a hypothetical accidental exposure that yields, for example, 141 dicentrics per 1303 metaphases, i.e., a yield of 0.11 dicentric per metaphase (this is the average assessment from the 10 volunteers), would be caused by an absorbed radiation dose of 1.00 (±0.05) Gy with a lower and upper 95% confidence limits, from combined Poisson and calibration curve errors on yield, of 0.90 and 1.10 Gy; respectively.

Acknowledging that the DCA is currently the best-established cytogenetic technique for absorbed radiation dose assessment, it is somewhat impractical for triage of mass casualty events owing to its labor-intensive and time-consuming procedures. While lymphocyte culture for 48 h is inevitable, many research is directed to streamline DCA processes by automating DC scoring in metaphase images using machine learning image processing techniques (36–40). Hence, many image training classifiers were created to improve the specificity of automated DC analysis algorithm. However, accuracy and efficiency of the automated (unsupervised) DC scoring are still requiring more optimization for accurate dose estimation (41). In this study, we have used the embedded Metafer DCScore algorithm to evaluate the usefulness of the automated DC score. Recognizing that the two calibrations curves obtained by the automated (unsupervised) and the assisted (supervised) dicentrics and metaphases scoring modes exhibited wide dissimilarities (Figure 5), results presented may reveal a potential use of the automated scoring mode for triage purposes (42). Of course, the relative overestimation of DC yields less than 1 Gy renders the reliability of automated (unsupervised) scoring at low radiation dose somewhat questionable and flawed with large portion of false-positive exposure. Furthermore, the steep underestimation greater than 2 Gy, along with the significant lack of goodness of fit (*P* < 0.0001), precludes the use of automated scoring for accurate dose estimation. However, providing a quick preliminary dose estimate can help decision making based on clinical signs in early triage of radiation casualties. Confirmation of results can follow after completing the assisted scoring.

The criteria for triage assumptions require performing the dose assessments on the analysis of as low as 50 metaphase spreads (43). The relevance is considered sufficient only for a preliminary assessment in a mass-casualty event. In general, the output of biodosimetry triage needed by physicians in a radiation emergency is to quickly place the victim into one of four dose ranges (1–2, 2–4, 4–6, and >6 Gy) to provide timely information for patient treatment planning (12). However, because of the uncertainty on triage dose estimation, it is probably more realistic to assign victims according to the following three categories adopted from Swartz et al. (44) with modifications:

- Category 1. Identify false-positives and near 2-Gy exposure: A triage-estimated dose less than 2 Gy may contain false- and true-positives low-radiation exposure who would not need urgent medical care. Those individuals can be discharged and followed later for accurate dose estimate and potential risks of long-term effects.
- Category 2. Exposure between 2 and 5 Gy: Those individuals require medical admission and observation for probable manifestation of hematopoietic ARS, which is curable with active medical care as needed.
- Category 3. Exposure greater than 5 Gy: This level identifies individuals with critical radiation exposure who may need intensive care.

The actual dose threshold level may vary with the associated trauma and the capacity of the healthcare system. The automated triage mode can provide valuable preliminary diagnostic evidence to segregate between these various categories of risk groups (41). Of particular significance, the automated mode can provide dose estimations that are close to those calculated by the assisted mode in the critical dose exposure range of 2 Gy, which can rapidly identify low risk victims (<2 Gy), who do not require urgent medical attention and high-risk victims (>2 Gy), requiring immediate medical countermeasures and potential therapeutic intervention.

## Conclusions

We have successfully established the reference DRCC for DC aberrations induced by X-rays of 320 kVp. There was no effect of age or sex groups in this cohort of 10 young adult volunteers. The national calibration curve obtained by the assisted (supervised) scoring of dicentric in Saudi Arabia was in the middle range of those described in other populations. Although the calibration curve obtained by the automated (unsupervised) scoring misrepresented dicentric yields at low and high doses, it can potentially be useful for triage mode to segregate between false-positives and near 2-Gy exposure and seriously irradiated individuals who require hospitalization. The biodosimetry laboratory is now participating in the evaluation of eventual accidental radiation exposures and contributing to the national preparedness plan in response to radiation emergencies. It can provide valuable information about the magnitude of radiation exposure to public health officials and decision-makers who evaluate the extent and consequence of public, medical, and occupational irradiation.

## Data Availability Statement

The original contributions presented in the study are included in the article/Supplementary Materials, further inquiries can be directed to the corresponding author.

## Ethics Statement

The studies involving human participants were reviewed and approved by The Basic Research and the Ethics Committees of the institutional review board (KFSHRC, RAC#2170 005). The patients/participants provided their written informed consent to participate in this study.

## Author Contributions

GAA and BAM: conceptualization and funding acquisition. GAA, KSA-H, and NMA-H: methodology. KSA-H, NMA-H, and SSBJ: investigation. GAA and KSA-H: formal analysis. GAA, BAM and KSA-H: validation. GAA: writing the original draft. All authors review and editing.

## Conflict of Interest

The authors declare that the research was conducted in the absence of any commercial or financial relationships that could be construed as a potential conflict of interest.
