# Supplementary material for: Establishing a Reference Dose–Response Calibration Curve for Dicentric Chromosome Aberrations to Assess Accidental Radiation Exposure in Saudi Arabia
Source: Front Public Health. 2020 Dec 15;8:599194. doi: 10.3389/fpubh.2020.599194 (PMC7793750; doi:10.3389/fpubh.2020.599194)
Supplement: Supplementary file 1 [file Table_1.DOCX]

**Supplementary Material 1:** Yield and intercellular distribution of dicentric chromosomal aberrations induced *in vitro* in peripheral blood lymphocytes by x-rays exposure. Results of 10 healthy Saudi blood volunteers.

| ***Dose (Gy)*** | ***N. metaphases*** | ***N. dicentrics*** | ***D0**** | ***D1**** | ***D2**** | ***D3**** | ***D4**** | ***D5**** | ***Y*** | ***DI*** | ***U-value*** |
| --- | --- | --- | --- | --- | --- | --- | --- | --- | --- | --- | --- |
| Volunteer 1: | | | | | | | | | | | |
| **0** | 2966 | 4 | 2962 | 4 | 0 | 0 | 0 | 0 | 0.001 | 1.00 | -0.04 |
| **0.1** | 1632 | 10 | 1622 | 10 | 0 | 0 | 0 | 0 | 0.006 | 0.99 | -0.17 |
| **0.25** | 1442 | 24 | 1418 | 24 | 0 | 0 | 0 | 0 | 0.017 | 0.98 | -0.44 |
| **0.5** | 1030 | 54 | 976 | 54 | 0 | 0 | 0 | 0 | 0.052 | 0.95 | -1.18 |
| **0.75** | 934 | 74 | 862 | 70 | 2 | 0 | 0 | 0 | 0.079 | 0.98 | -0.52 |
| **1** | 714 | 104 | 614 | 96 | 4 | 0 | 0 | 0 | 0.146 | 0.93 | -1.28 |
| **2** | 436 | 195 | 280 | 118 | 37 | 1 | 0 | 0 | 0.447 | 0.97 | -0.51 |
| **3** | 364 | 322 | 163 | 98 | 86 | 16 | 1 | 0 | 0.885 | 0.99 | -0.17 |
| **4** | 348 | 485 | 87 | 120 | 90 | 28 | 14 | 9 | 1.394 | 1.04 | 0.59 |
| **5** | 250 | 511 | 28 | 81 | 56 | 39 | 29 | 17 | 2.044 | 0.98 | -0.19 |
| Volunteer 2: | | | | | | | | | | | |
| **0** | 3820 | 7 | 3813 | 7 | 0 | 0 | 0 | 0 | 0.002 | 1.00 | -0.07 |
| **0.1** | 2307 | 16 | 2291 | 16 | 0 | 0 | 0 | 0 | 0.007 | 0.99 | -0.23 |
| **0.25** | 1623 | 27 | 1597 | 25 | 1 | 0 | 0 | 0 | 0.017 | 1.06 | 1.69 |
| **0.5** | 1238 | 33 | 1207 | 29 | 2 | 0 | 0 | 0 | 0.027 | 1.10 | 2.41 |
| **0.75** | 1046 | 64 | 986 | 56 | 4 | 0 | 0 | 0 | 0.061 | 1.06 | 1.49 |
| **1** | 771 | 89 | 688 | 77 | 6 | 0 | 0 | 0 | 0.115 | 1.02 | 0.41 |
| **2** | 497 | 186 | 345 | 121 | 28 | 3 | 0 | 0 | 0.374 | 1.03 | 0.41 |
| **3** | 376 | 318 | 147 | 153 | 65 | 9 | 2 | 0 | 0.846 | 0.81 | -2.60 |
| **4** | 293 | 372 | 73 | 108 | 80 | 25 | 6 | 1 | 1.270 | 0.81 | -2.25 |
| **5** | 226 | 441 | 27 | 59 | 72 | 45 | 12 | 11 | 1.951 | 0.82 | -1.95 |
| Volunteer 3: | | | | | | | | | | | |
| **0** | 3032 | 10 | 3022 | 10 | 0 | 0 | 0 | 0 | 0.003 | 1.00 | -0.12 |
| **0.1** | 1876 | 12 | 1864 | 12 | 0 | 0 | 0 | 0 | 0.006 | 0.99 | -0.19 |
| **0.25** | 1678 | 28 | 1650 | 28 | 0 | 0 | 0 | 0 | 0.017 | 0.98 | -0.47 |
| **0.5** | 1222 | 56 | 1168 | 52 | 2 | 0 | 0 | 0 | 0.046 | 1.03 | 0.66 |
| **0.75** | 1086 | 67 | 1020 | 65 | 1 | 0 | 0 | 0 | 0.062 | 0.97 | -0.73 |
| **1** | 684 | 76 | 612 | 68 | 4 | 0 | 0 | 0 | 0.111 | 1.00 | -0.08 |
| **2** | 470 | 172 | 323 | 124 | 21 | 2 | 0 | 0 | 0.366 | 0.95 | -0.77 |
| **3** | 310 | 244 | 142 | 102 | 58 | 6 | 2 | 0 | 0.787 | 0.94 | -0.78 |
| **4** | 282 | 342 | 76 | 124 | 50 | 16 | 10 | 6 | 1.213 | 1.07 | 0.78 |
| **5** | 248 | 484 | 27 | 83 | 56 | 52 | 17 | 13 | 1.952 | 0.89 | -1.26 |
| Volunteer 4: | | | | | | | | | | | |
| **0** | 3231 | 6 | 3225 | 6 | 0 | 0 | 0 | 0 | 0.002 | 1.00 | -0.07 |
| **0.1** | 1750 | 10 | 1740 | 10 | 0 | 0 | 0 | 0 | 0.006 | 0.99 | -0.16 |
| **0.25** | 1202 | 20 | 1182 | 20 | 0 | 0 | 0 | 0 | 0.017 | 0.98 | -0.40 |
| **0.5** | 994 | 46 | 950 | 42 | 2 | 0 | 0 | 0 | 0.046 | 1.04 | 0.94 |
| **0.75** | 944 | 62 | 884 | 58 | 2 | 0 | 0 | 0 | 0.066 | 1.00 | 0.00 |
| **1** | 596 | 86 | 515 | 77 | 3 | 1 | 0 | 0 | 0.144 | 1.00 | -0.05 |
| **2** | 480 | 155 | 359 | 88 | 32 | 1 | 0 | 0 | 0.323 | 1.13 | 2.03 |
| **3** | 280 | 200 | 130 | 110 | 32 | 6 | 2 | 0 | 0.714 | 0.91 | -1.08 |
| **4** | 214 | 268 | 58 | 74 | 62 | 12 | 6 | 2 | 1.252 | 0.90 | -1.02 |
| **5** | 220 | 412 | 13 | 98 | 58 | 22 | 13 | 16 | 1.873 | 0.89 | -1.17 |
| Volunteer 5: | | | | | | | | | | | |
| **0** | 4722 | 5 | 4717 | 5 | 0 | 0 | 0 | 0 | 0.001 | 1.00 | -0.05 |
| **0.1** | 2101 | 13 | 2088 | 13 | 0 | 0 | 0 | 0 | 0.006 | 0.99 | -0.19 |
| **0.25** | 1544 | 19 | 1525 | 19 | 0 | 0 | 0 | 0 | 0.012 | 0.99 | -0.33 |
| **0.5** | 1191 | 30 | 1162 | 28 | 1 | 0 | 0 | 0 | 0.025 | 1.04 | 1.05 |
| **0.75** | 1094 | 48 | 1048 | 44 | 2 | 0 | 0 | 0 | 0.044 | 1.04 | 0.95 |
| **1** | 883 | 83 | 805 | 73 | 5 | 0 | 0 | 0 | 0.094 | 1.03 | 0.58 |
| **2** | 455 | 177 | 312 | 115 | 23 | 4 | 1 | 0 | 0.389 | 1.08 | 1.16 |
| **3** | 406 | 282 | 211 | 128 | 52 | 11 | 3 | 1 | 0.695 | 1.11 | 1.56 |
| **4** | 308 | 389 | 70 | 131 | 77 | 19 | 8 | 3 | 1.263 | 0.83 | -2.11 |
| **5** | 247 | 486 | 27 | 76 | 65 | 48 | 19 | 12 | 1.968 | 0.86 | -1.57 |
| Volunteer 6: | | | | | | | | | | | |
| **0** | 3318 | 14 | 3304 | 14 | 0 | 0 | 0 | 0 | 0.004 | 1.00 | -0.17 |
| **0.1** | 2460 | 20 | 2440 | 20 | 0 | 0 | 0 | 0 | 0.008 | 0.99 | -0.28 |
| **0.25** | 2290 | 24 | 2266 | 24 | 0 | 0 | 0 | 0 | 0.010 | 0.99 | -0.35 |
| **0.5** | 1392 | 48 | 1345 | 46 | 1 | 0 | 0 | 0 | 0.034 | 1.01 | 0.21 |
| **0.75** | 958 | 42 | 916 | 42 | 0 | 0 | 0 | 0 | 0.044 | 0.96 | -0.95 |
| **1** | 880 | 64 | 818 | 60 | 2 | 0 | 0 | 0 | 0.073 | 0.99 | -0.19 |
| **2** | 456 | 152 | 333 | 95 | 27 | 1 | 0 | 0 | 0.333 | 1.06 | 0.96 |
| **3** | 308 | 234 | 148 | 98 | 51 | 10 | 1 | 0 | 0.760 | 0.99 | -0.16 |
| **4** | 232 | 298 | 68 | 70 | 64 | 22 | 6 | 2 | 1.284 | 0.97 | -0.34 |
| **5** | 218 | 430 | 32 | 58 | 60 | 38 | 12 | 18 | 1.972 | 1.01 | 0.14 |
| Volunteer 7: | | | | | | | | | | | |
| **0** | 2968 | 6 | 2962 | 6 | 0 | 0 | 0 | 0 | 0.002 | 1.00 | -0.07 |
| **0.1** | 1636 | 12 | 1624 | 12 | 0 | 0 | 0 | 0 | 0.007 | 0.99 | -0.20 |
| **0.25** | 1270 | 30 | 1240 | 30 | 0 | 0 | 0 | 0 | 0.024 | 0.98 | -0.59 |
| **0.5** | 1142 | 40 | 1102 | 40 | 0 | 0 | 0 | 0 | 0.035 | 0.97 | -0.83 |
| **0.75** | 934 | 70 | 865 | 68 | 1 | 0 | 0 | 0 | 0.075 | 0.95 | -0.99 |
| **1** | 648 | 86 | 566 | 79 | 2 | 1 | 0 | 0 | 0.133 | 0.99 | -0.27 |
| **2** | 470 | 182 | 321 | 121 | 24 | 3 | 1 | 0 | 0.387 | 1.04 | 0.67 |
| **3** | 408 | 302 | 188 | 162 | 38 | 16 | 4 | 0 | 0.740 | 0.99 | -0.13 |
| **4** | 282 | 391 | 83 | 76 | 82 | 22 | 10 | 9 | 1.387 | 1.14 | 1.68 |
| **5** | 242 | 446 | 38 | 69 | 71 | 33 | 19 | 12 | 1.843 | 0.97 | -0.30 |
| Volunteer 8: | | | | | | | | | | | |
| **0** | 4666 | 11 | 4655 | 11 | 0 | 0 | 0 | 0 | 0.002 | 1.00 | -0.11 |
| **0.1** | 3238 | 29 | 3209 | 29 | 0 | 0 | 0 | 0 | 0.009 | 0.99 | -0.35 |
| **0.25** | 1832 | 45 | 1787 | 45 | 0 | 0 | 0 | 0 | 0.025 | 0.98 | -0.74 |
| **0.5** | 2676 | 101 | 2577 | 97 | 2 | 0 | 0 | 0 | 0.038 | 1.00 | 0.08 |
| **0.75** | 1395 | 94 | 1306 | 85 | 3 | 1 | 0 | 0 | 0.067 | 1.06 | 1.62 |
| **1** | 2552 | 254 | 2307 | 238 | 5 | 2 | 0 | 0 | 0.100 | 0.99 | -0.45 |
| **2** | 1361 | 475 | 961 | 339 | 49 | 10 | 2 | 0 | 0.349 | 1.03 | 0.91 |
| **3** | 1232 | 901 | 578 | 481 | 108 | 57 | 7 | 1 | 0.731 | 1.00 | 0.10 |
| **4** | 619 | 767 | 174 | 257 | 91 | 64 | 29 | 4 | 1.239 | 1.06 | 1.03 |
| **5** | 466 | 881 | 71 | 159 | 89 | 72 | 47 | 28 | 1.891 | 1.08 | 1.22 |
| Volunteer 9: | | | | | | | | | | | |
| **0** | 3812 | 4 | 3808 | 4 | 0 | 0 | 0 | 0 | 0.001 | 1.00 | -0.04 |
| **0.1** | 2100 | 10 | 2090 | 10 | 0 | 0 | 0 | 0 | 0.005 | 1.00 | -0.15 |
| **0.25** | 1950 | 22 | 1928 | 22 | 0 | 0 | 0 | 0 | 0.011 | 0.99 | -0.34 |
| **0.5** | 1420 | 54 | 1367 | 52 | 1 | 0 | 0 | 0 | 0.038 | 1.00 | -0.01 |
| **0.75** | 1324 | 98 | 1228 | 94 | 2 | 0 | 0 | 0 | 0.074 | 0.97 | -0.84 |
| **1** | 908 | 123 | 792 | 110 | 5 | 1 | 0 | 0 | 0.135 | 1.00 | -0.09 |
| **2** | 450 | 163 | 307 | 124 | 18 | 1 | 0 | 0 | 0.362 | 0.90 | -1.54 |
| **3** | 342 | 270 | 148 | 132 | 52 | 8 | 0 | 2 | 0.789 | 0.92 | -0.99 |
| **4** | 290 | 390 | 67 | 123 | 60 | 21 | 11 | 8 | 1.345 | 1.04 | 0.46 |
| **5** | 168 | 326 | 18 | 65 | 32 | 26 | 16 | 11 | 1.940 | 1.00 | 0.04 |
| Volunteer 10: | | | | | | | | | | | |
| **0** | 5419 | 9 | 5410 | 9 | 0 | 0 | 0 | 0 | 0.002 | 1.00 | -0.08 |
| **0.1** | 5120 | 24 | 5096 | 24 | 0 | 0 | 0 | 0 | 0.005 | 1.00 | -0.23 |
| **0.25** | 4958 | 78 | 4881 | 76 | 1 | 0 | 0 | 0 | 0.016 | 1.01 | 0.51 |
| **0.5** | 4017 | 163 | 3856 | 159 | 2 | 0 | 0 | 0 | 0.041 | 0.98 | -0.71 |
| **0.75** | 4652 | 314 | 4344 | 303 | 4 | 1 | 0 | 0 | 0.067 | 0.98 | -1.10 |
| **1** | 4392 | 443 | 3973 | 400 | 15 | 3 | 1 | 0 | 0.101 | 1.03 | 1.63 |
| **2** | 3137 | 1003 | 2272 | 732 | 128 | 5 | 0 | 0 | 0.320 | 0.97 | -1.36 |
| **3** | 1619 | 1125 | 819 | 553 | 183 | 52 | 10 | 2 | 0.695 | 1.05 | 1.44 |
| **4** | 1648 | 1873 | 537 | 579 | 363 | 119 | 39 | 11 | 1.137 | 1.00 | 0.01 |
| **5** | 762 | 1398 | 126 | 238 | 174 | 120 | 68 | 36 | 1.835 | 1.03 | 0.57 |

N. metaphases: number of cells in metaphase assessed. N. dicentrics: total number of dicentrics found in the metaphases assessed. *Number of metaphases with 0, 1, 2, 3, 4, 5 dicentrics, respectively. Y: yield of dicentrics, i.e. the number of dicentrics per metaphase (cell). DI: Dispersion index. U-value: a u-value between -1.96 and +1.96 indicates a Poisson distribution.
